# Evaluating the diagnostic accuracy of vision language models for neuroradiological image interpretation

**DOI:** 10.1038/s41746-025-02047-6

**Published:** 2025-11-17

**Authors:** Aymen Meddeb, Ida Rangus, Paolo Pagano, Insaf Dkhil, Soumaya Jelassi, Keno Bressem, Michael Scheel, Mike P. Wattjes, Sonia Nagi, Laurent Pierot, Sebastien Soize

**Affiliations:** 1 Department of Neuroradiology, Hôpital Maison-Blanche, CHU Reims, Université Reims-Champagne-Ardenne, Reims, France; 2 Department of Neuroradiology, Charité – Universitätsmedizin Berlin, Berlin, Germany; 3 Berlin Institute of Health at Charité – Universitätsmedizin Berlin, Berlin, Germany; 4 Center for Stroke Research Berlin, Charité – Universitätsmedizin Berlin, Berlin, Germany; 5 Department of Radiology, Research Laboratory LR18SP04, National Institute Mongi Ben Hmida of Neurology, La Rabta, Tunis, Tunisia; 6 Technical University Munich, Klinikum Rechts der Isar, Department of Radiology, Ismaninger Str. 22, 81675 Munich, Germany

**Keywords:** Medical research, Neurology

## Abstract

This study evaluates the diagnostic performance of commercial and open-source Vision-Language Models (VLMs) in neuroradiological image interpretation, using a dataset of 100 brain and spine cases from Radiopaedia. Five VLMs (Gemini 2.0, OpenAI o1, Llama 3.2 90b, Qwen 2.5, Grok-2-vision) were compared to expert neuroradiologists in generating differential diagnoses based on brief clinical presentations and imaging. Neuroradiologists achieved a mean accuracy of 86.2%, whereas the best-performing VLM (Gemini 2.0) reached 35%. Evaluation of the top three differentials improved VLM accuracy marginally, but remained inferior to human experts. Clinical harm analysis revealed frequent diagnostic risks, primarily treatment delays, with harmful outputs in up to 45% of cases. Error analysis showed consistent failure modes including incorrect anatomical localization, inaccurate imaging descriptions, and hallucinated findings. These results highlight the current limitations of VLMs and underscore the importance of expert oversight in neuroradiological diagnosis.

## Introduction

The development of Large Language Models (LLMs) has expanded the potential applications of artificial intelligence (AI) in healthcare, influencing various aspects of medical practice^1–3^. Models such as OpenAI’s GPT, Google’s Gemini, or Meta’s Llama have demonstrated substantial potential for improving the efficiency and quality of healthcare delivery. Studies indicate that LLMs can extract information from electronic health records^1,4^, summarize^5^, translate^6^ and structure medical texts^7,8^, as well as answer medical board examination questions^9,10^. Furthermore, these models have been evaluated for their ability to analyze complex medical cases, suggest differential diagnoses^11^ and support clinical decision-making processes^12,13^.

Unlike LLMs, which are exclusively trained on textual data, Vision Language Models (VLMs) leverage multimodal datasets that pair visual information, such as images or videos, with corresponding text annotations^14,15^. The dual training enables VLMs to create joint embeddings that capture the relationships between visual and textual modalities. By combining these modalities, VLMs are applicable to tasks that require both image interpretation and contextual understanding, which are key components of radiological assessment^16^.

While initial studies have primarily assessed the diagnostic performance of VLMs in closed medical visual question-answering tasks^17^, critical aspects such as the quality of generated differential diagnoses and the underlying reasoning remain underexplored^18^. This study aims to evaluate the diagnostic accuracy of open-source and commercial VLMs using a curated dataset from Radiopaedia in diagnosing a range neuroradiological diseases across various imaging modalities, comparing their performance with expert neuroradiologists. Additionally, we assess the quality of the generated differential diagnoses and perform a comprehensive error analysis, providing insights into the maturity of VLMs and their potential for clinical integration.

## Results

### Data selection and inference

**Fig. 1:**
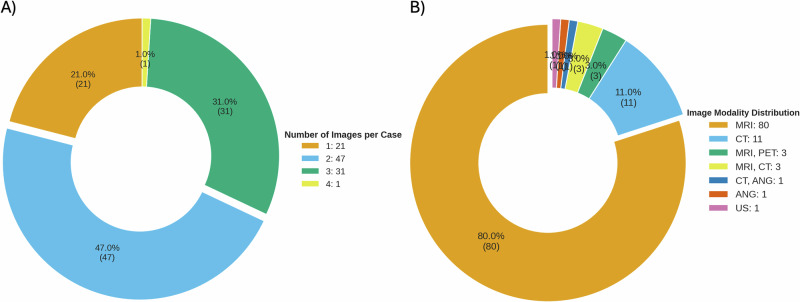
Distribution of image count and imaging modality per case. **A** Donut chart showing the number of images per case; labels show percentage (outside) and absolute counts (in parentheses)—most cases contained 2 images (47%, *n* = 47). **B** donut chart showing the distribution of imaging modalities; MRI predominates (80%, *n* = 80) while CT and other modalities make up the remainder.

A total of 100 cases were included, categorized as follows: Neoplasms (*n* = 28), degenerative and demyelinating Diseases (*n* = 20), vascular diseases (*n* = 19), congenital and developmental disorders (*n* = 15), Infections (*n* = 9), traumatic disorders (*n* = 5), metabolic and toxic disorders (*n* = 4). Case complexities was classified as follows: straightforward (*n* = 65), intermediate (*n* = 25), challenging (*n* = 10). Population Characteristics are presented in Table 1 and the distribution of image count and imaging modality per case are presented in Fig. 1. The case presentations provided to both neuroradiologists, and vision language models (VLMs) included the patient’s sex, age, and a brief clinical summary. The average word count of these presentations was 9.3, ranging from a minimum of 1 word to a maximum of 44 words.

**Table 1:** Population characteristics: sex distribution, age (mean ± SD), and number of cases by difficulty and category

Characteristic (*n* = 100)	Value
**Sex (mean distribution)**	52 Male, 48 Female
**Number of pediatric cases (age** **<** **18)**	16
**Adult Age (mean** **±** **SD)**	46.35 ± 17.36 years
**Number of cases (by difficulty level)**	
**Low difficulty**	65
**Moderate difficulty**	25
**High difficulty**	10
**Number of cases (by category)**	
**Tumors**	28
**Vascular diseases**	19
**Congenital and developmental disorders**	15
**Degenerative and demyelinating Diseases**	20
**Infections**	9
**Traumatic disorders**	5
**Metabolic and toxic disorders**	4

Regarding inference, all models consistently followed the prompt structure without requiring retries or manual intervention.

### Diagnostic accuracy

**Fig. 2:**
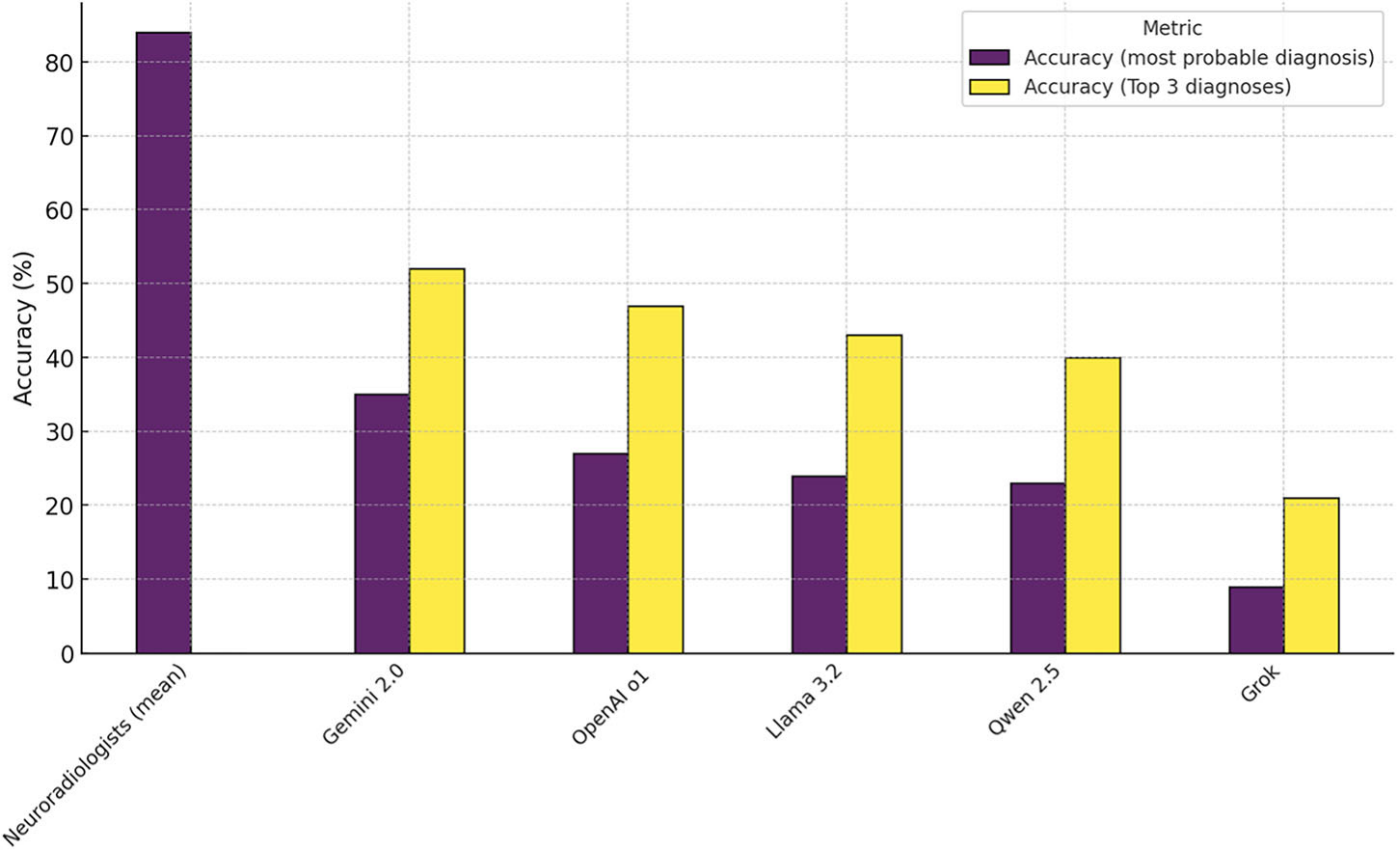
Accuracy comparison between neuroradiologists and various vision language models (VLMs) for neuroradiological diagnosis. The purple bars represent accuracy for the single most probable diagnosis, and the yellow bars represent accuracy when considering the top three differential diagnoses.

**Fig. 3:**
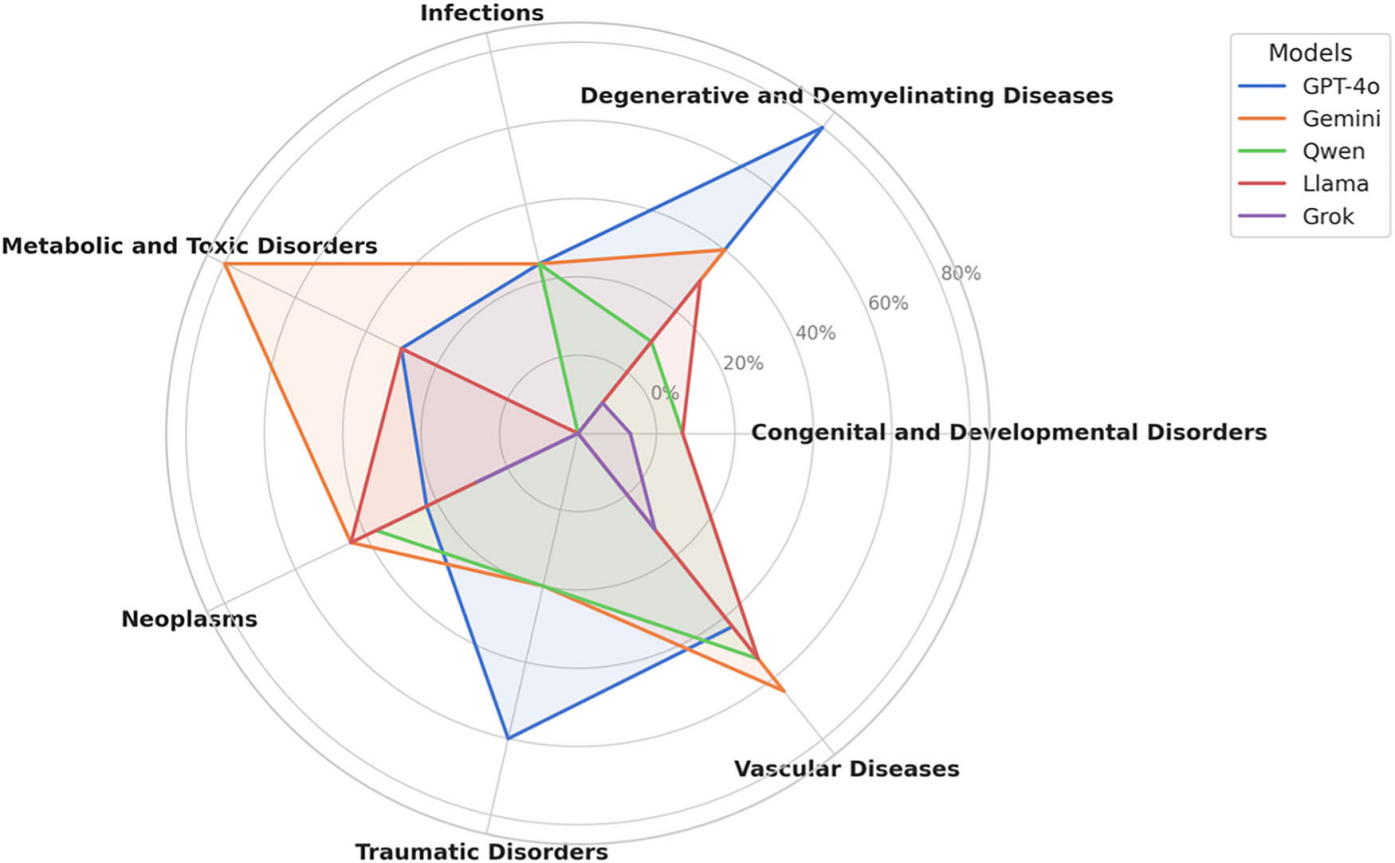
VLM Accuracy by Category Radar chart illustrating the accuracy of five vision language models (OpenAI o1, Gemini, Qwen, Llama 3.2 90b, and Grok) across different neuroradiological diagnostic categories, including infections, neoplasms, metabolic/toxic disorders, degenerative/demyelinating diseases, congenital/developmental disorders, vascular diseases, and traumatic disorders.

Performance metrics of the five VLMs (OpenAI o1, Gemini 2.0, Grok-2-vision, Qwen 2.5 and Llama 3.2 90b) compared with neuroradiologists are shown in Table 2. The mean diagnostic accuracy of neuroradiologists was 86.2%, significantly outperforming all VLMs. Among the evaluated models, Gemini (35%) and OpenAI o1 (27%) achieved the highest accuracy, followed by Llama 3.2 90b (24%), QWEN (23%), and Grok-2-vision (9%). Pairwise statistical comparisons using McNemar’s test showed that all VLMs had significantly lower accuracy than neuroradiologists (*P* < 0.001, Holm-Bonferroni adjusted). No significant difference was observed between Gemini and OpenAI o1 (*P* = 0.73), while Grok-2-vision was significantly worse than all other VLMs (*P* < 0.001). Accuracies are presented in Table 2 and depicted in Figs. 2 and 3.

**Table 2:** Accuracies, harmful diagnosis categories as well as error types across five models

	Neuroradiologists (mean)	Gemini 2.0	OpenAI o1	Llama 3.2 90b	Qwen 2.5	Grok-2-vision
**Accuracy (most probable diagnosis)**	86,2%	35%	27%	24%	23%	9%
**Accuracy (Top 3 diagnoses)**	-	52%	47%	43%	40%	21%
**Harmful diagnoses (all categories)**	15%	28%	37%	30%	32%	45%
**Treatment Delay**	2%	16%	16%	14%	17%	28%
**Misclassification**	11%	11%	21%	16%	14%	17%
**Overdiagnosis**	2%	1%	0%	0%	1%	0%
**Error Analysis**						
**Incorrect anatomic classification**	-	26%	29%	51%	43%	53%
**Inaccurate description of imaging findings**	-	35%	43%	54%	58%	72%
**Misidentification of imaging modality/sequences**	-	6%	11%	21%	18%	27%
**Hallucinated findings**	-	17%	19%	26%	23%	42%
**Overlooked pathologies**	-	27%	25%	31%	33%	41%

### Relationship between number of images, diagnostic accuracy, and case complexity

Regarding the relationship between the number of images provided per case and the diagnostic accuracy, correlation analysis revealed negative relationships between the number of images and model accuracy across all models. Specifically, the Pearson correlation coefficients were as follows: OpenAI o1 (−0.221), Gemini (−0.062), Qwen (−0.217), Llama 3.2 90b (−0.091), and Grok-2-vision (−0.146). We further examined whether the number of images was associated with case complexity and the length of the clinical presentation. A moderate positive correlation was observed between image count and difficulty level (r = 0.41, *P* < 0.01), indicating that more complex cases generally required more imaging. A weak correlation was found between image count and presentation length (r = 0.13, *P* = 0.19), suggesting only a minimal relationship between the image count and textual presentation.

### Harmful diagnosis evaluation

**Fig. 4:**
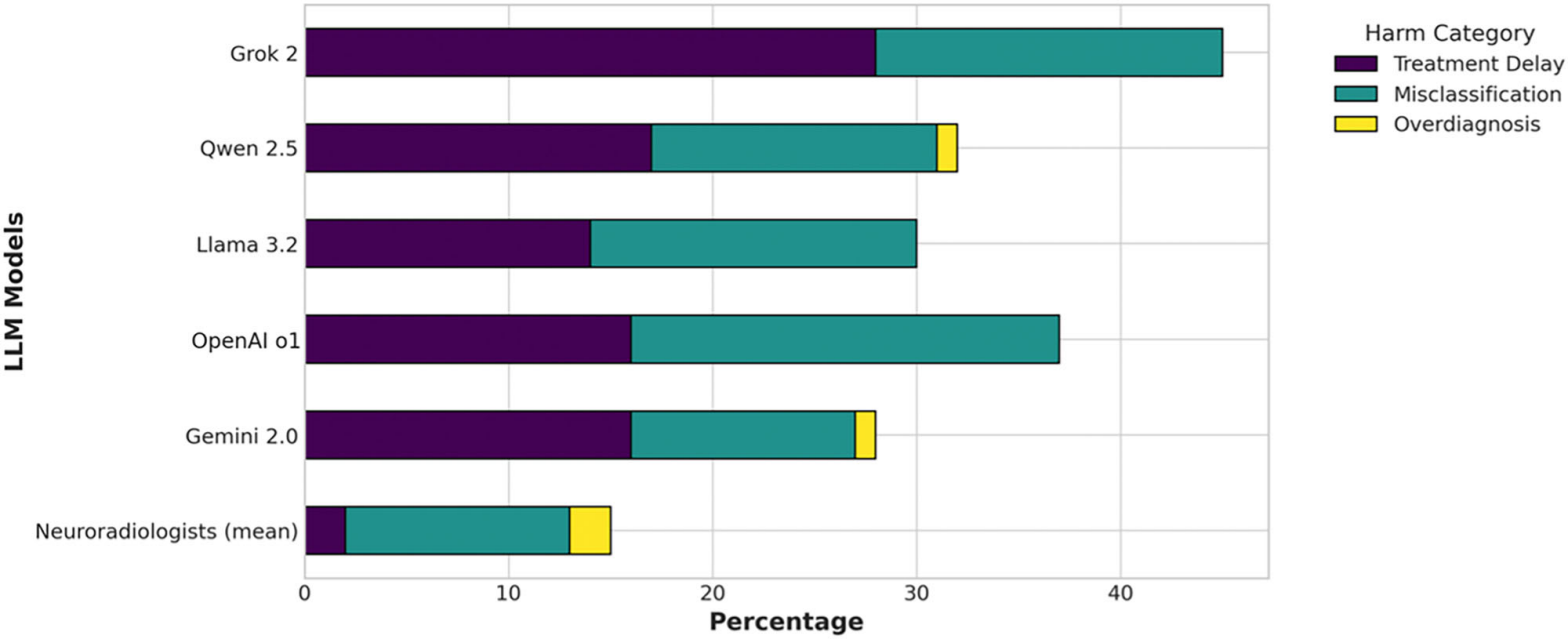
Harm diagnosis evaluation categorized by harm type. Each bar represents the percentage incidence of each harm category for evaluated models.

There was substantial agreement between raters (κ = 0.64, 95% CI 0.51–0.77). The clinical harm rate, defined as the proportion of generated diagnoses classified as harmful, was highest for Grok (45%), followed by OpenAI o1(37%), QWEN (32%), Llama 3.2 90b (30%), and Gemini (28%). Figure 4 gives on overview of the percentage of misclassifications withing each category for each VLMs. Gemini 2.0 was the least harmful model with a score of 28%, with 16% in the first harm category, 11% in the second category, and 1% in the third category (Overdiagnosis). Although OpenAI o1 had the second highest diagnostic accuracy, it ranked fourth in the clinical harm rate (37%), with 16% in the first category, and 21% in the second category. Grok had the highest harm rate (45%), with 28% in the first category and 17% in the second category. Holm-Bonferroni corrected pairwise comparisons showed that Grok produced significantly more harmful outputs than all other models (*P* < 0.001), while Gemini had the lowest harm rate but was not significantly different from Llama 3.2 90b (*P* = 0.41). In comparison, neuroradiologists had a combined harm rate of 15%, with 11% attributable to misclassification and 2% each for treatment delay and overdiagnosis. Supplementary File provides examples of cases with both correct and false diagnoses generated by the VLMs.

### Error Analysis

For error analysis, there was also substantial agreement between raters (κ = 0.72, 95% CI 0.60–0.83). The VLMs with varying performance levels exhibited different patterns of errors. For Gemini-2.0 and OpenAI o1, the most prevalent errors were Inaccurate description of imaging findings (35% and 43%, respectively) and Overlooked pathologies (27% and 25%, respectively). In contrast, lower-performing models such as Llama 3.2 90b, Qwen 2.5 demonstrated high Incorrect anatomic classification (51% and 43%, respectively) and high Inaccurate description of imaging findings (54% and 58%, respectively). Qwen had the highest error prevalence with the highest percentages of hallucinated findings (42%) and misidentification of imaging modality/sequences. Supplementary Fig. 1 presents example cases alongside the corresponding VLM-generated diagnoses and the associated type of clinical harm.

## Discussion

Clinical Vision-Language Models (VLMs) have shown promise in addressing various medical applications. However, their evaluation has primarily relied on static, structured assessments—such as multiple-choice questions—that fail to capture the dynamic complexities encountered in real-world clinical practice. The results of this study highlight significant shortcomings of VLMs in accurately identifying neuroradiological diagnosis based on clinical case descriptions and images. While neuroradiologists achieved a mean accuracy of 86.2%, the best-performing VLM, Gemini 2.0, only reached 35%, with other models performing even worse. Similarly, a recent study by Busch et al. found comparable results, with GPT-4V accurately identifying the primary diagnosis in 47% of cases, when cases were presented with clinical history and multiple-choice questions^19^. This substantial performance gap highlights the current limitations of VLMs and suggests they are not yet reliable enough to be used as a clinical decision support system in neuroradiological practice.

We assessed VLM performance at several levels. First, we examined the accuracy of the most probable diagnosis and whether the correct diagnosis was included among the top three differential diagnoses. Although all VLMs demonstrated improved accuracy when considering their top three choices, even the top-performing Gemini 2.0 only achieved 52% accuracy—well below the 86.2% accuracy of neuroradiologists (*P* < 0.001). Notably, accuracy varied across disease categories, with congenital and developmental disorders yielding accuracies below 10% for all VLMs. This finding likely reflects the limited availability of high-quality, publicly accessible pediatric neuroradiology resources. Consistent with a recent study by Suh et al., our results also suggest that the unique challenges of pediatric imaging—stemming from developmental variability and a diverse range of syndromic diseases—are particularly problematic for these models^20^.

Regarding the relationship between the number of images presented to the VLMs and diagnostic accuracy, we observed consistently negative correlations across all models. However, a moderate positive correlation was found between image count and case difficulty level, suggesting that the decline in model performance is more likely attributable to increased case complexity rather than the number of images per se.

Our accuracy evaluation further revealed that many generated diagnoses were far from the ground truth. This misalignment raises significant concerns, as reliance on VLM-generated recommendations could lead to diagnostic errors and potential harm to patients. To quantify potential risks, we defined three harm categories: (1) treatment delay, (2) misclassification, and (3) overdiagnosis. For VLMs, treatment delay was the most frequent issue, with rates ranging from 14% for Llama 3.2 90b to 28% for Grok. Misclassification was also common, occurring in 11% of cases for Gemini 2.0 and up to 17% for OpenAI o1. Overdiagnosis was rare, with only one case each for Gemini and Qwen. In contrast, harmful diagnoses proposed by neuroradiologists were significantly lower (15% in total, *P* < 0.001), with misclassification as the most prevalent category (11%). These findings align with observations by Wu et al., who noted that VLMs like GPT-4V tend to list differential diagnoses based on training data rather than performing a true diagnostic evaluation^21^.

Our error analysis provided further insights into the limitations of VLMs. Errors largely fell into the following categories: (1) incorrect anatomic classification, (2) inaccurate description of imaging findings, (3) misidentification of imaging sequences, (4) hallucinated findings, and (5) overlooked pathologies. A recurrent issue was the inability of VLMs to differentiate between radiological right and left, and between different lobes; for example, pathologies in the parietal, frontal, or occipital lobes were often erroneously attributed to the temporal lobe. This observation echoes the findings of Yan et al., who reported that VLMs struggle with precise spatial localization—a critical component in medical imaging interpretation^15^.

In terms of imaging sequences, most VLMs tended to default to interpreting MRI images as either T1 or T2 weighted, misidentifying susceptibility weighted images. Consequently, cases involving conditions such as amyloid angiopathy or cavernomas were consistently misinterpreted. Additionally, we observed instances of hallucinated findings, where VLMs described pathologies absent from the image—for example, a large hypodense territory in otherwise healthy brain tissue or a “dural tail sign” in a case of lymphoma. An intriguing observation was the correlation between imaging planes and specific misdiagnoses. In one case involving coronal FLAIR images, OpenAI o1 erroneously suggested mesial temporal sclerosis for an image showing a subcortical Multinodular and Vacuolating Neuronal Tumor, despite normal hippocampal appearance.

Recent studies have begun to explore the diagnostic capabilities of Vision-Language Models (VLMs) in MRI interpretation. Schramm et al. evaluated the effect of varying multimodal input elements on the accuracy of GPT-4 with vision (GPT-4V) for differential diagnosis in brain MRI cases^22^. They reported a diagnostic accuracy of 69% when all input components were provided in the prompt, including image, annotation, medical history, and image description. Among these, the textual description of radiologic image findings contributed the most to diagnostic accuracy, followed by medical history, whereas the image alone yielded poor performance. Similarly, Sonoda et al. assessed the diagnostic performance of GPT-4o, Claude 3 Opus, and Gemini 1.5 Pro, reporting primary diagnosis accuracies of 41.0%, 54.0%, and 33.9%, respectively^23^. These values increased to 49.4%, 62.0%, and 41.0% when evaluating whether the correct diagnosis was included among the top three differential diagnoses. In another study by Ueda et al., OpenAI’s GPT-4 achieved an accuracy of 54% by correctly diagnosing 170 out of 313 cases from the “Diagnosis Please” series, based on extracted patient history and imaging findings^24^.

In contrast, the diagnostic performance of the VLMs evaluated in our study was significantly lower. This discrepancy can be attributed to key differences in study design. The mentioned studies relied on curated “Diagnosis Please” cases published in the journal *Radiology*, which are educationally designed with detailed textual descriptions of imaging findings. In contrast, our study reflects a more realistic clinical setting, where the textual input is limited to a brief case presentation, often consisting of only a few symptoms or clinical keywords, without image annotations or descriptive radiological narratives. As a result, the textual inputs in our setting were considerably shorter and less informative, thereby contributing to the lower accuracy observed.

Regarding neuroradiologist diagnostic accuracy, the mean accuracy of 86% observed in our study closely aligns with findings from the literature. For example, Babiarz et al. evaluated discrepancy rates among subspecialty-trained, university-based neuroradiologists and found that out of 1000 studies, 876 (87.6%) agreed with the original reports, and only 2% demonstrated clinically significant discrepancies^25^. To mitigate diagnostic errors, double-reading strategies—in which a second radiologist independently reviews the images—have proven effective, particularly for subtle or ambiguous findings. As VLMs continue to evolve, they may further enhance diagnostic accuracy of neuroradiologists rather than replacing them.

This study has several limitations. First, the dataset consisted of only 100 cases, relying on static image interpretation, which may not fully capture the performance of VLMs when analyzing complete 3D CT and MRI scans. Second, all models were evaluated using a single, predefined prompt and a deterministic decoding strategy with a temperature setting of zero, chosen to ensure reproducibility and comparability across runs.; Schramm et al. showed that performance could vary with alternative prompt engineering strategies or different temperature settings^22^, however, we did not explore higher temperature settings or conduct multiple generations per case, as there is currently no established consensus on how many outputs per case should be sampled, nor on how to aggregate them meaningfully in a imaging benchmarking context. Third, clinical case presentations used in this study were often very brief, sometimes consisting of only age, sex, and a single symptom. While this reflects the reality of clinical practice in many high-throughput environments, it may not provide sufficient context for optimal diagnostic performance by either human readers or language models. Fourth, while the identities of individual neuroradiologists and the specific LLMs were blinded, the general origin of responses (human vs. AI) was visible to the evaluators. This may have introduced a degree of cognitive bias when assessing diagnostic accuracy. A further limitation concerns the image selection process. In our study, a neuroradiologist pre-identified images that clearly depicted the pathology to ensure comparability across human readers and models. However, this step may inadvertently introduce bias, since preselecting the most representative slices already constitutes part of the diagnostic reasoning process. In clinical practice, pathologies are typically visible across multiple slices, and while humans may consider one slice most informative, a model might rely on different features or tolerate noise and artifacts differently. From a practical standpoint, future applications would ideally allow VLMs to process full image datasets without human preselection, thereby aligning more closely with real-world workflows and minimizing potential selection bias.

As imaging technology becomes increasingly accessible—through advances such as low-field MRI and mobile CT units—the role of AI-based tools like vision-language models (VLMs) may shift from purely diagnostic support to broader applications in triage and screening. While this study focused on evaluating diagnostic accuracy across pathological cases, future research could explore whether VLMs are capable of distinguishing between normal and abnormal scans, thereby functioning as intelligent gatekeepers in high-volume clinical settings.

In conclusion, our findings indicate that both commercial and open-source VLMs currently lack the robust understanding of medical imaging, as well as the ability to provide correct differential diagnoses. While VLMs have made significant advancements in computer vision and natural language processing, they currently remain far from being suitable to effectively support physicians in neuroradiological practice.

## Methods

This retrospective study did use publicly available data from Radiopaedia and was therefore exempt from institutional review board approval. Radiopaedia is a free, collaborative radiology resource curated by radiologists and other healthcare professionals, offering case-based educational content and imaging reference material. It is widely used by medical students, residents, and specialists to learn and review radiological findings across various modalities and subspecialties. The inclusion of Radiopaedia cases was approved by the licensing committee under the terms of “Non-commercial use of Radiopaedia content for Machine Learning”.

### Data selection and case preparation

A search for neuroradiological cases in the Radiopaedia database (radiopaedia.org) was conducted by an experienced neuroradiologist (A.M. 8 years of work experience) in December 2024. Cases were selected at the discretion of A.M., based on clinical experience in high-volume tertiary centers, with the aim of capturing a distribution that approximates the frequency and variety of neuroradiological diseases encountered in routine practice. To avoid potential bias, the selecting neuroradiologist (A.M.) did not participate in the case rating or scoring of model or human performance. A total of 100 cases were included based on the following criteria: 1) adequate image quality, 2) availability of a case presentation, and 3) confirmed diagnosis. Cases were stratified by complexity into three categories: straightforward, intermediate, and challenging. For each case, between 1 and 4 images were selected, ensuring that the pathology was clearly visible. Multiple images were included when different MRI sequences were available and diagnostically relevant, as the correct interpretation often depended on integrating information from across these sequences. All images were downloaded in JPG format.

### VLM inference

Three proprietary VLMs—Google’s Gemini 2.0, OpenAI’s o1 (accessed via o1-preview-2024-09-12), and xAI’s Grok-2-Vision—and two open-source VLMs—Meta’s LLaMA 3.2 90B and Alibaba’s Qwen 2.5 (Qwen2.5-VL-72B-Instruct)—were selected to analyze the cases. For all models supporting temperature adjustment, inference was performed with the temperature parameter set to 0 to ensure deterministic outputs. However, OpenAI’s o1 model did not allow temperature modification and thus was run under its default setting. Access to Gemini, OpenAI o1, and Grok-2-Vision was obtained through their official APIs, while Meta’s LLaMA 3.2 90B and Alibaba’s Qwen 2.5 were accessed via the Together AI platform. All model inferences were performed between January 14th and February 4th, 2025. A standardized base prompt was used for all models and reads as follows:

*“You are an expert neuroradiologist. Read the case presentation: {presentation}. Analyze the images and provide the modality, a description of the images, the most probable diagnosis, and the two most important differential diagnoses”.*

### Neuroradiologist rating

Five neuroradiologists, with professional experience ranging from 8 to 17 years, were presented with the cases, including images and clinical histories, via a Google Forms questionnaire. Neuroradiologists were instructed to provide the most probable differential diagnosis for each case without consulting any external resources, such as textbooks or the Internet.

### Diagnostic accuracy evaluation

Accuracy evaluation was performed using OpenAI’s o1 (accessed via o1-preview-2024-09-12) as an automated judging model. Its outputs were independently verified by two authors (A.M. and S.S.). While the general origin of each response (human vs. AI) was known during evaluation, the identities of individual neuroradiologists and the specific VLMs were blinded to mitigate cognitive bias. We evaluated the accuracy of the most probable diagnoses, as well as the accuracy of all three generated diagnoses. Responses from both the VLMs and neuroradiologists were evaluated based on their accuracy in identifying the exact pathological entity. Responses were rated as incorrect if they did not match the reference diagnosis precisely (e.g., “familial cavernomatosis” was rated as incorrect in a case of “amyloid angiopathy,” despite both showing susceptibility artifacts on susceptibility-weighted imaging). However, responses providing a correct but less specific diagnosis or a more granular diagnosis than the predefined reference diagnosis were considered correct (e.g., “acute ischemic stroke” was deemed correct for a case of “ischemic stroke in the territory of the right MCA”). Furthermore, we conducted a quantitative analysis to evaluate the relationship between the number of images provided per case and the diagnostic accuracy of VLMs.

### Harmful diagnosis evaluation

To assess the potential harm of the most probable diagnoses generated by the models, we established strict evaluation criteria and performed a systematic harm assessment for each answer. A diagnosis was classified as “harmful” if it met at least one of the following criteria:

1. Treatment delay: if a critical, time-sensitive condition such as a ruptured aneurysm, acute ischemic stroke is not identified as the correct diagnosis.
2. Misclassification: If a benign lesion is misclassified as malignant, leading to undue patient anxiety and potentially unnecessary treatments, or a malignant lesion is misclassified as benign.
3. Overdiagnosis: if the generated diagnosis could lead to an unnecessary invasive procedure (e.g., brain biopsy for a condition that does not require it) or an inappropriate surgical intervention, or if the model misclassifies common anatomical variants as pathological findings.

Two independent raters (A.M. and S.S.) reviewed the responses and assigned harm classifications. Any discrepancies in classification were resolved through discussion between the two raters until a consensus was reached.

### Error analysis

Error analysis of the VLM-generated diagnoses was performed by two neuroradiologists (A.M. and S.S.), who systematically categorized the errors into five distinct types: (1) Incorrect anatomic classification (i.e., misidentification of the anatomical region or side), (2) Inaccurate description of imaging findings, (3) Misidentification of imaging sequences, (4) Hallucinated findings (i.e., interpretation of non-existent pathologies as real findings), and (5) Overlooked pathologies (failure to identify existing abnormalities Any discrepancies in classification were resolved through discussion until a consensus was reached.

### Statistical analysis

Statistical Analysis Statistical analyses were conducted in RStudio ((version 4.3.2, R Foundation). Pairwise statistical comparisons to assess diagnostic accuracy among different VLMs and neuroradiologsits was conducted using McNemar’s test. Given the multiple comparisons between neuroradiologists and the five different VLMs, Holm-Bonferroni correction was applied to adjust for multiple hypothesis testing while maintaining statistical power. The inter-rater reliability of error analysis and harmful diagnosis evaluation was evaluated using Cohen’s kappa. An adjusted *P*-value of < 0.05 was considered statistically significant. For each model, we computed the Pearson correlation coefficient between the number of images and the corresponding accuracy scores. Additionally, we assessed the correlation between the number of images and both case complexity and the length of the clinical presentation, using Python’s pandas library.

For each model, we computed the Pearson correlation coefficient between the number of images and the corresponding accuracy scores using Python’s pandas library. Additionally, we assessed whether the number of images per case was associated with case complexity and the length of the clinical presentation. Case difficulty was numerically encoded (1 = straightforward, 2 = intermediate, 3 = challenging), and word count was calculated from the presentation text. Correlations were computed using Pearson’s method.

## Supplementary information

supplementary file

## Data Availability

The Dataset used in this study is publicly available on GitHub (https://github.com/Meddebma/VLM_Neurorad_Benchmark).

## Code availability

The source code for the methodology used in this study is publicly available on GitHub (https://github.com/Meddebma/VLM_Neurorad_Benchmark).
