# Supplementary material for: Evaluating the diagnostic accuracy of vision language models for neuroradiological image interpretation
Source: NPJ Digit Med. 2025 Nov 17;8:666. doi: 10.1038/s41746-025-02047-6 (PMC12623885; doi:10.1038/s41746-025-02047-6)
Supplement: Supplementary file 1 — supplementary file [file 41746_2025_2047_MOESM1_ESM.pdf]

Images	Case presentation and ground truth	Gemini 2.0	GPT-4o1-Preview	Llama 3.2	Qwen 2.5	Grok
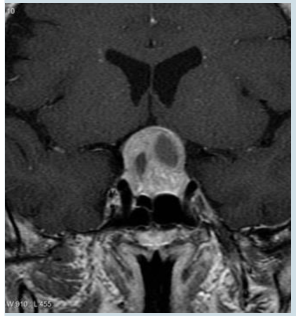 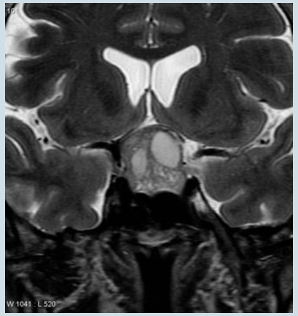	75 y.o. female with Visual failure. Diagnosis: **Pituitary macroadenoma**	Pituitary Adenoma	Pituitary Adenoma	Pituitary Adenoma	Pituitary Adenoma	Pituitary Adenoma
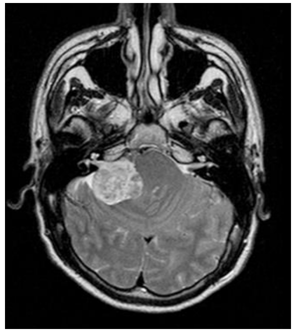 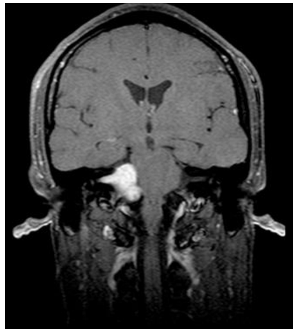	47 y.o. female with Sensorineural hearing loss. Diagnosis: **Vestibular schwannoma**	Vestibular schwannoma	Vestibular schwannoma	Vestibular schwannoma	Vestibular schwannoma	Vestibular schwannoma
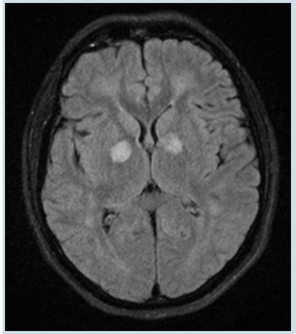 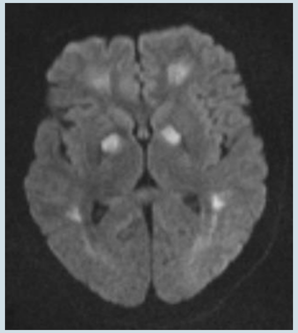	30 y.o., previously healthy man went into a coma after exposure to a heating gas in a closed environment. Diagnosis: **Carbon Monoxide Poisoning**	Carbon Monoxide Poisoning	Carbon Monoxide Poisoning	Carbon Monoxide Poisoning	Neurotoxicity-related injury Harm type: Treatment Delay	Acute ischemic stroke. Harm type: Misclassification

Images	Case presentation and ground truth	Gemini 2.0	GPT-4o1-Preview	Llama 3.2	Qwen 2.5	Grok
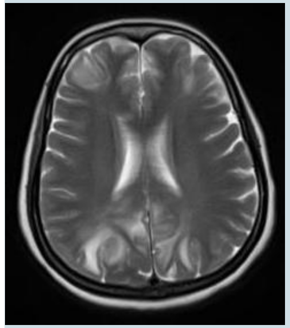 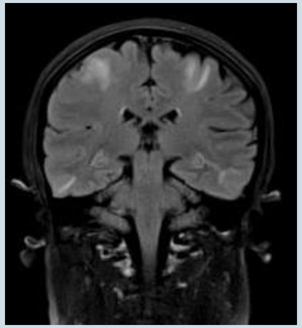	32 y.o. postpartum hypertensive female, presented with headache and seizures. Diagnosis: **Posterior reversible encephalopathy syndrome (PRES)**	severe pre-eclampsia/eclampsia with associated posterior reversible encephalopathy syndrome (PRES)	Posterior Reversible Encephalopathy Syndrome (PRES)	Normal pressure hydrocephalus (NPH) Harm type: Treatment Delay	Hypertensive Intracranial Hemorrhage Harm type: Treatment Delay	Multiple Sclerosis (MS) Harm type: Treatment Delay
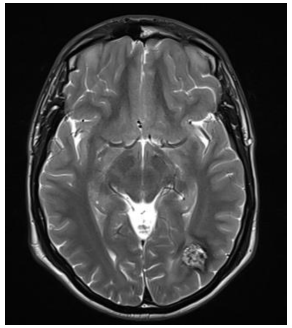 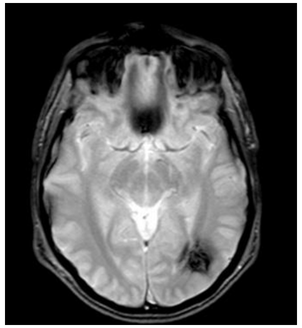	32 y.o. male with seizures (episodes of blackout lasting for 30 minutes associated with headache). 6 attacks over the last 5 months. Diagnosis: **Cavernoma**	high-grade glioma Harm type: Misclassification	Low-grade glioma Harm type: Misclassification	Tumor in the left parietal cortex Harm type: No Harm	Glioblastoma multiforme (GBM) Harm type: Misclassification	Glioblastoma multiforme (GBM) Harm type: Misclassification
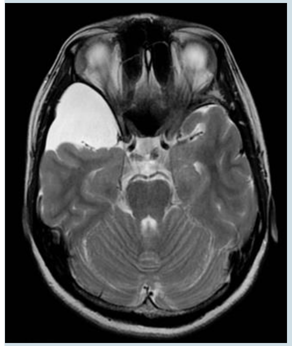 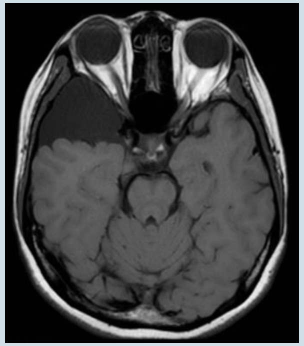	28 y.o. female, presented with headaches Diagnosis: **Arachnoid cyst**	Meningioma Harm type: Overdiagnosis	Meningioma Harm type: Overdiagnosis	Epidermoid cyst Harm type: Misclassification	Meningioma Harm type: Overdiagnosis	Optic nerve glioma Harm type: Misclassification

Supplementary Figure 1. *Examples of VLM diagnoses compared to ground truth across clinical cases*.

Each row presents a neuroradiological case including two representative MR images, the clinical case presentation with the ground truth diagnosis, and the output diagnosis from five Vision-Language Models (Gemini 2.0, GPT o1, Llama 3.2, Qwen 2.5, Grok; right). Green highlights indicate a correct diagnosis matching the ground truth. Red highlights indicate incorrect diagnoses, with associated harm types shown below when applicable.
